# Exploring factor XIII genetic diversity: a familial approach to inheritance and variation

**DOI:** 10.1186/s12959-025-00766-0

**Published:** 2025-11-13

**Authors:** Arshi Naz, Sana Zameer, Hyder Ali Pehilwani Rind, Tehmina Nafees Sonia Khan, Younus Jamal Siddiqi, Abdul Rehman Khalil Shaikh, Shahida Memon, Ikram din Ujjan, Eva katona, László Muszbek

**Affiliations:** 1 Liaquat University of Medical and Health Sciences, Jamshoro, Sindh Pakistan; 2 Baqai Medical University, Karachi, Sindh Pakistan; 3 IMU University, Kuala Lumpur, Malaysia; 4 Jinnah Medical and Dental College, Karachi, Sindh Pakistan; 5 University of Debrecen, Debrecen, Hungary

**Keywords:** Factor XIII, Rare inherited bleeding disorder, Autosomal recessive trait, Gene mutation, Familial disease, Consanguinity

## Abstract

**Background:**

Plasma coagulation factor XIII (OMIM#134570 (F13A1) and 134580(F13B), synthesized in haematopoietic cells (FXIII-A) and hepatocytes (FXIII-B); stabilizes and protects fibrin clots against fibrinolytic breakdown, ensuring haemostasis. Inherited FXIII deficiency is a rare inherited autosomal recessive bleeding disorder affecting 1–3 million people globally and demonstrating strong consanguinity contributing to high incidence of cases in Pakistan. Patients manifesting severe illness are homozygotes or compound heterozygotes.

**Aims:**

This study aims to estimate phenotypic traits, genetic alterations, and carrier rates in families with known genetic abnormalities in individuals with Factor XIII deficiency.

**Methods:**

This cross-sectional study was approved by Advanced Studies Research Board and Ethical Review Committee of LUMHS, Jamshoro and conducted in concordance with Declaration of Helsinki 2000 in collaboration at the Biochemistry Department of LUMHS and Haematology Department, Baqai medical university, Karachi. Written informed consent obtained from all participants included in the study. Pedigree was constructed. Direct DNA sequencing performed via big dye terminator by using selective exon as per previously identified mutations in the patients of their families. FXIII confirmed with clot solubility testing and Elisa performed for Assay antigen detection for FXIII. Pathogenicity scoring done by using different software.

**Results:**

All the families had a history of consanguineous marriages and history of bleeding. From the six families, four families show same mutation in patient i.e. IVS11 (+ 1) G > A while two families showed c.2045G > A mutation in their homozygous patient.

**Conclusion:**

The results of this study highlight how crucial it is to combine biochemical, clinical, and statistical approaches to increase the precision of diagnoses, improve patient treatment, and make genetic counselling easier for families who are at risk.

## Introduction

Coagulation factor XIII is a protransglutaminase that plays a significant role in the terminal phase of blood coagulation [1].

**Fig. 1:**
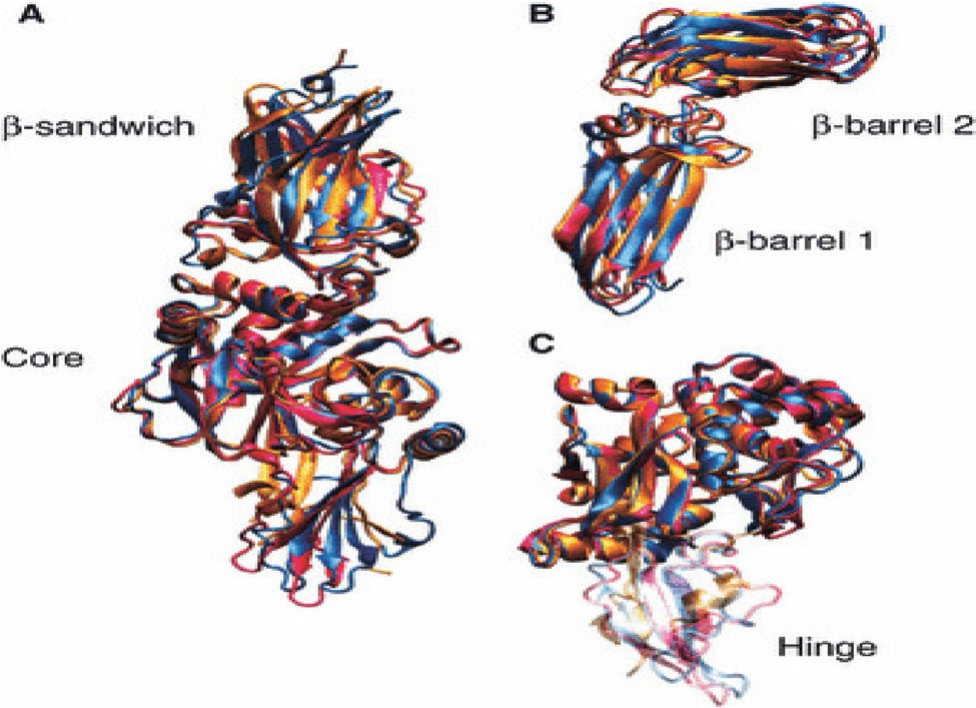
Illustrates The β-sandwich, β-barrel, core, and hinge sections of Factor XIII are highlighted in this picture, which depicts its intricate structural arrangement. Its ability to stabilize blood clots by crosslinking fibrin depends on these structural characteristics. (Komáromi, I., Bagoly, Z. and Muszbek, L. (2011) [3]

Despite being uncommon, factor XIII insufficiency is a serious bleeding illness that presents many difficulties for those who have it as well as their families. Deficits in the A or B subunits of Factor XIII are the outcome of this autosomal recessive disorder, which is brought on by mutations in the F13A1 or F13B genes. The 160 kb gene that codes for the FXIII-A subunit (F13A) is found on chromosome 6p24-25. The FXIII-B subunit gene (F13B) is found on chromosome 1q31-32.1. It is around 28 kb long and is made up of 12 exons that are broken up by 11 introns. These exons encode the mature 641 amino acid protein. Because of their form, the ten tandem repeats that make up the FXIII B subunit are known as glycoprotein-1 structures or sushi domains [2] (Fig. 1).

Clinical indications of the condition range from minor symptoms to serious, potentially fatal problems, such as repeated miscarriages, poor wound healing, and spontaneous bleeding. Improving the quality of life and lowering the morbidity and mortality linked to this illness depend on early diagnosis and treatment. Finding carriers, who are usually asymptomatic and have genetic mutation, is still an important but little-studied part of treating Factor XIII insufficiency [2, 4].

Understanding the genetic transmission of the condition depends on carrier identification, which also has important ramifications for genetic counselling and reproductive decision-making. Healthcare professionals can provide more informed advice by identifying carriers in the families of known Factor XIII-deficient patients, empowering at-risk individuals to take proactive measures to avoid illness and prepare for family planning. Because autosomal recessive illnesses like Factor XIII insufficiency are more common in communities with high consanguinity rates, this strategy is particularly crucial. In addition to reducing the disorder’s intergenerational transmission, carrier identification is crucial for the early diagnosis and treatment of any possible problems in subsequent generations [5, 6].

In order to improve illness care and prevention, this study highlights the need to identify carriers in the families of patients with Factor XIII deficiency. To clarify inheritance patterns, carrier frequency, and the range of mutations linked to Factor XIII insufficiency, it seeks to examine the genetic and clinical characteristics of such families. The study also looks at the wider effects of carrier status on reproductive choices, family dynamics, and psychological health. In doing so, it draws attention to how important it is to incorporate genetic counselling and screening programs into healthcare systems, especially in under-resourced areas [7, 8].

To avoid sickness and improve the quality of life for impacted families, this thesis seeks to emphasize the significance of finding carriers in known Factor XIII-deficient families. It looks at the usefulness of diagnostic instruments, assesses the difficulties in putting carrier screening into practice in environments with limited resources, and suggests solutions. The project aims to support international efforts to battle uncommon bleeding diseases, encourage early intervention, and improve health outcomes for impacted populations by expanding understanding in this field [5].

## Methods

### Research design

This study employed a cross-sectional design in compliance with the ethical principles outlined in the Helsinki Declaration (2000). Prior to commencement, the study protocol was reviewed and approved by the Advanced Studies Research Board and the Ethical Review Committee of Liaquat University of Medical and Health Sciences (LUMHS), Jamshoro. The reporting of this observational research adheres to the STROBE (Strengthening the Reporting of Observational Studies in Epidemiology) guidelines to ensure methodological transparency and rigor. The research was conducted at the Department of Biochemistry, LUMHS, in collaboration with the Haematology Department of Baqai Medical University (Karachi), as well as the Diagnostic& Research Laboratory (Hyderabad) and Dr. Arshi Diagnostic Laboratory (Karachi).

### Study environment and data collection

**Fig. 2:**
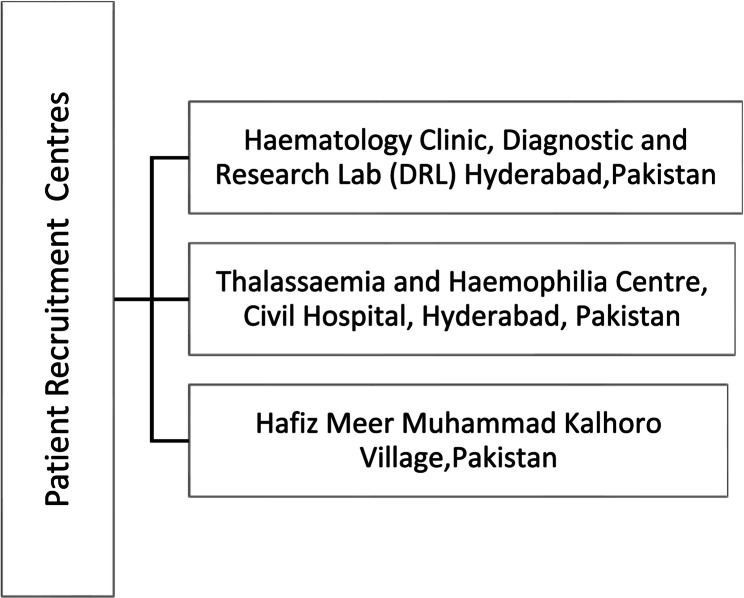
Illustrates the details of study cases of FXIII in Sindh province

Family members of known genetic defects of f.xiii deficient patients were residents of Hafiz Meer Muhammad Kalhoro village, Dadu, Kandiaro and Pathan Colony, Hyderabad. All these patients were registered in haematology clinic, Diagnostic & Research lab and Thalassaemia/Haemophilia Centre in Hyderabad shown in Fig. 2.

**Fig. 3:**
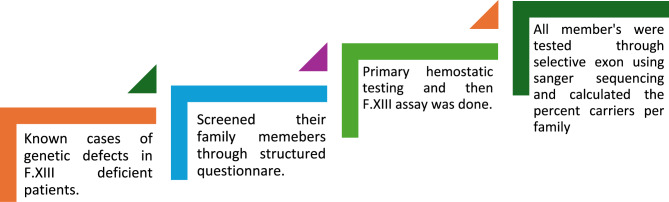
Expression of sequential analysis of study participants

Furthermore, collaborated on performing the sample processing in Diagnostic and Research lab, Hyderabad and processing in Dr Arshi Diagnostic Lab, Karachi, Pakistan. The sequential analysis of patients in the study is elaborated in Fig. 3.

Total 187 participants were included in this study (90 females and 97 males) with the IQR 27.31 years (age range 1 - 85 years). The study duration was 18 months (March 2023 to September 2024).

### Inclusion and exclusion

All the cases of other inherited bleeding disorders and bleeding secondary to other causes were excluded. Written informed consent was obtained from all individual participants included in the study in local languages Urdu and Sindhi. The subjects were interviewed face-to-face at their respective centres mentioned above.

using a standardized open-ended structured questionnaire. A qualified medical professional and supervisor conducted the interview after consent.

Bleeding score was calculated using ISTH BAT Score (https://www.mdcalc.com/calc/10580/isth-scc-bleeding-assessment-tool) [9]

### Specimen collection and transportation

For coagulation and molecular testing, peripheral blood samples were obtained in EDTA (Ethylenediaminetetraacetic acid) and sodium citrated tubes [cat no. 373083; Becton, Dickinson)] according to a predetermined protocol: Via two 3.0 ml tubes, draw venous blood and add 0.3 ml of 3.2% sodium citrate and 2 ml of K2 EDTA 7.2 mg (lavender top) (catalogue # 367841; BD Vacutainer^®^). The samples were drawn, transported via maintaining cold chain to transfer and stored in Diagnostic & research lab further processing in Dr Arshi Diagnostic Lab, Karachi.

### Specimen processing and storage

Prothrombin time (PT), activated partial thromboplastin time (aPTT), and complete blood count (CBC) were done to analyse the samples. The CBC was performed using the XN-1000 haematology analyser (Sysmex, Japan). Prior to testing, EDTA containing samples were mixed by gentle inversions 8–10 times.

The isolated Platelet Poor Plasma (PPP) was utilized for coagulation tests. Aliquots of platelet-poor plasma were preserved for the coagulation profile, which included the PT, aPTT, urea clot solubility testing by using 5 M urea solution and F-XIII assayed by ELISA(Enzyme Linked Immunosorbent Assay) method as per instructions of package insert using proper calibration and commercial control plasma. The coagulation tests were conducted on the STA Compact Analyzer (Diagnostic Stago, S.A.S, France).

### Molecular analysis

For molecular testing, the EDTA tubes were kept between 2 °C and 8 °C. In a chilled centrifuge, sodium citrated tubes were spun for a minimum of 10 min at 3500 rpm, at 4 °C. DNA Extraction from Peripheral Blood: The QiaAmp DNA Blood micro kit (Qiagen^®^ Hilden Gmbh) was used to extract genomic DNA. The Qubit^®^ 2.0 flourometer (Life Technology^®^) was used to determine the concentration and purity of DNA. A fluorescence-based tool called a fluorometer is used to measure DNA, RNA, and proteins with extreme precision and sensitivity.

DNA was quantified using the Qubit dsDNA HS (High Sensitive) test kit, according to the manufacturer’s instructions.

The Big dye terminator cycle sequencing kit (version 3.1) was used to do direct gene sequencing of the whole F13 gene using di-deoxy terminator cycle sequencing chemistry, which was then sequenced using an automated genetic analyser ABI 3500 (Applied Biosysteminc, USA). Selective exons were run as per mutations previously identified in the patients of their families.

## Results

This study presents detailed demographic, clinical, and statistical insights that contribute to a deeper understanding of the factors affecting carrier identification in families with FXIII deficiency.

**Fig. 4:**
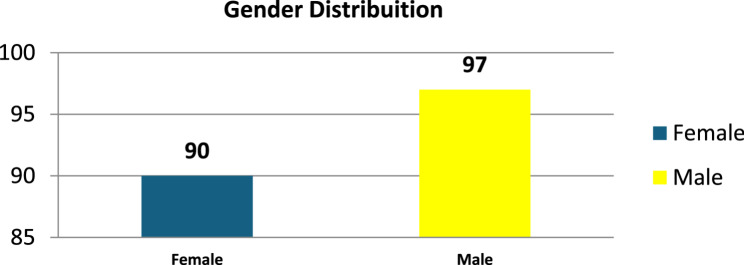
Shows the gender distribution in participants of the study. With 187 participants, mean age of ±27.31 years and age ranging from 1 to 85 years, the population is primarily young people. A comparison of carrier status and bleeding tendencies across generations is made possible by the broad age range, which implies the inclusion of several generations. An estimation of the mean age is shown by the comparatively low standard error (1.1914)

The gender distribution for 187 participants of this study shown in Fig. 4.

**Fig. 5:**
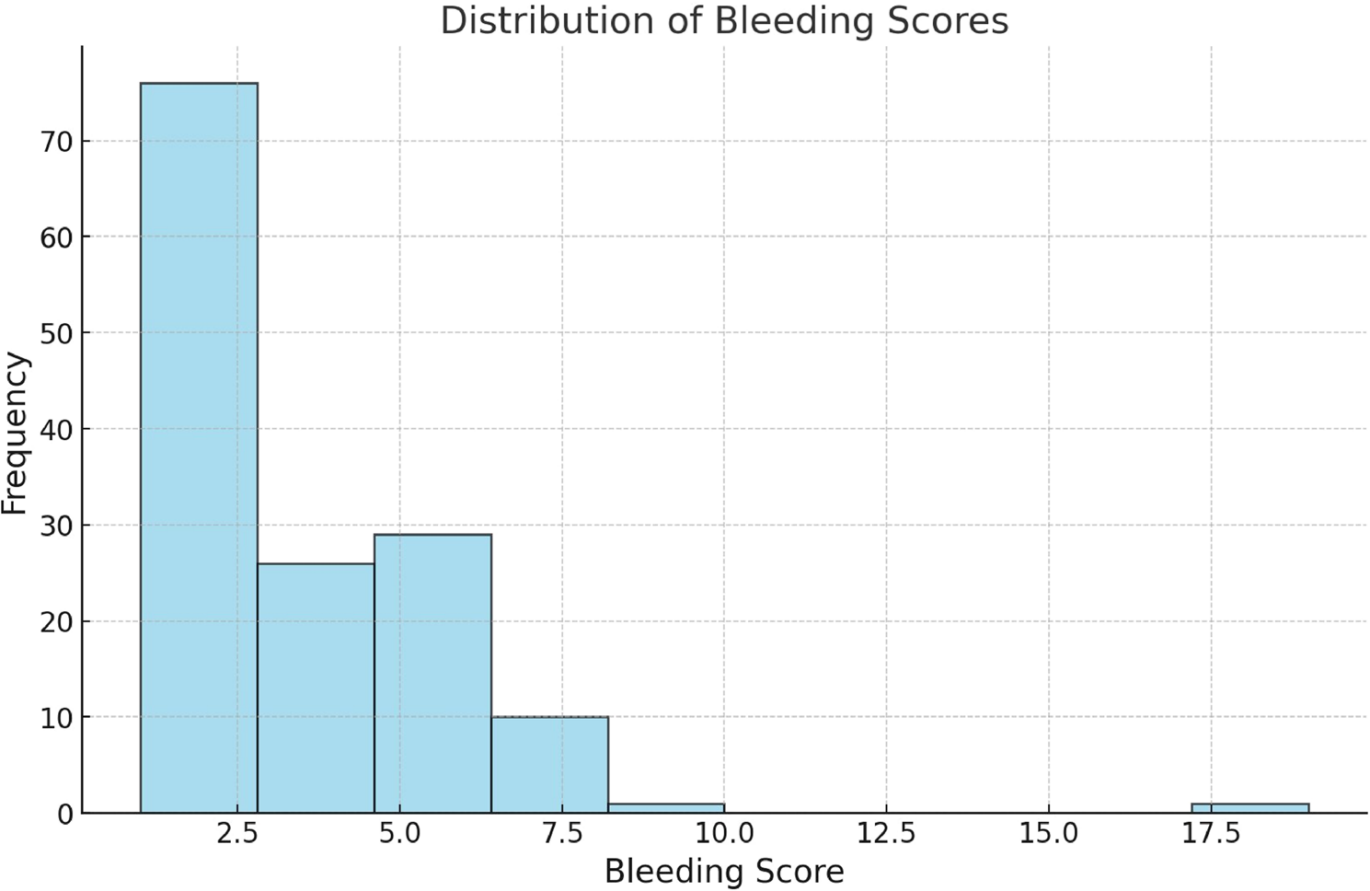
The histogram shows a right-skewed distribution of bleeding scores, with most scores concentrated between 2.5 and 5.0. Few individuals have scores above 10.0, indicating low overall bleeding scores in the sample

Bleeding scores using ISTH BAT assessment score, ranges from 1 to 19, reflecting variability from mild to severe haemorrhage. Moderate variance (7.051) and standard deviation (2.6554) indicate stable bleeding patterns despite most individuals having mild symptoms. (Figure 5).

**Fig. 6:**
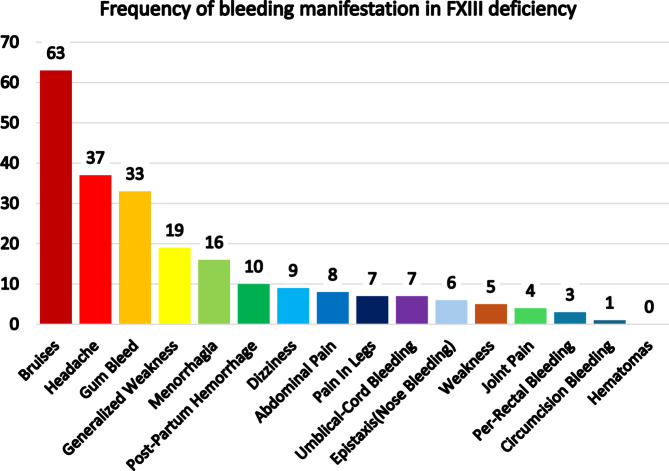
The bar graph shows frequency of bleeding manifestation in FXIII deficiency

As per frequency of bleeding symptoms, cutaneous bruises were the most frequent observed symptom in 63 participants out of 187. The second most common bleeding symptom was gum bleeding which was recorded in 33 participants. The sixteen female participants (18%) out of 90 females had menorrhagia. While one male participant out of 95 was reported to have post circumcision bleeding. (Figure 6).

**Fig. 7:**
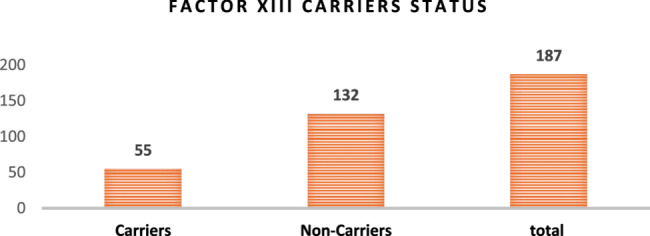
The bar graph illustrates the distribution of Factor XIII carrier status in a population, highlighting a significantly larger group of non-carriers (*N*=132) compared to carriers (*N*=55). This indicates that non-carriers are more prevalent in the studied sample

Significant disparities were also noted in FXIII activity between carriers (*N*=55) and non-carriers(*N*=132) shown in Fig. 7.

**Fig. 8:**
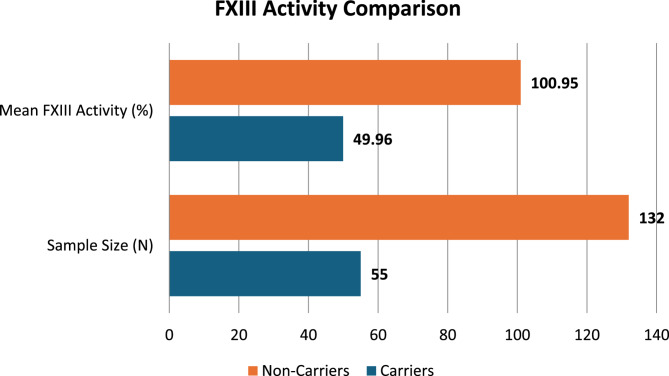
The bar graph compares FXIII activity between carriers and non-carriers, showing significantly higher mean FXIII activity (100.95%) and sample size (*N*=132) for non-carriers compared to carriers (49.96% and *N*=55)

The mean FXIII activity in carriers was 49.96%, which is lower than the 100.95% observed in non-carriers. This discrepancy highlights the different metabolic characteristics of carriers. A more uniform deficit pattern among carriers is shown by the much lower standard deviation for carriers (26.27%) in comparison to non-carriers (79.22%). Greater accuracy in predicting the mean FXIII activity of carriers is indicated by the decreased standard error (3.75% for carriers vs. 6.92% for non-carriers). (Figure 8).

A statistically significant difference in FXIII activity between carriers and non-carriers is shown by a one-way ANOVA (*F* = 19.44, *p*=.000), which further supports these findings. The variance in FXIII activity is mostly due to variations between groups rather than within-group variability, as indicated by the high F-value and low *p*-value. This result is further supported by the significant sum of squares between groups (92,736.42) as opposed to within groups (849,043.64).

### Pedigree construction

**Fig. 9:**
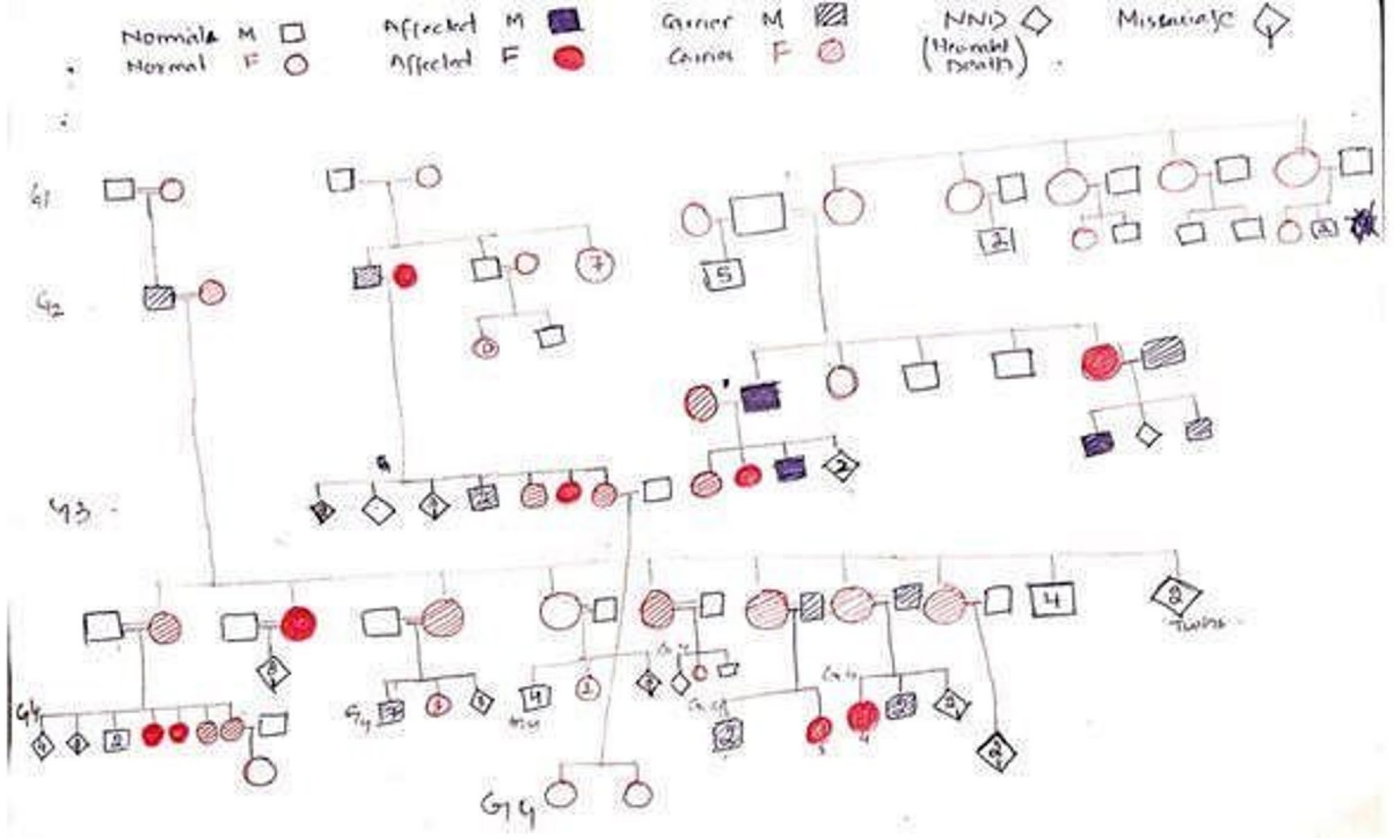
Pedigree of the families that are included in this study

Our study identified 10 homozygous patients from six different families. All the families had a history of consanguineous marriages and history of bleeding (Fig. 9).

From the six families, four families show same mutation in patient i.e IVS11 (+1) G>A while two families showed c.2045G>A mutation in their homozygous patient.

The results also highlight how crucial bleeding scores are as an additional diagnostic tool to FXIII activity, providing a thorough method of carrier identification. This study highlights the potential of FXIII activity levels to enhance early diagnosis, direct family planning, and inform focused treatments in families impacted by Factor XIII insufficiency by combining clinical, statistical, and demographic data.

**Fig. 10:**
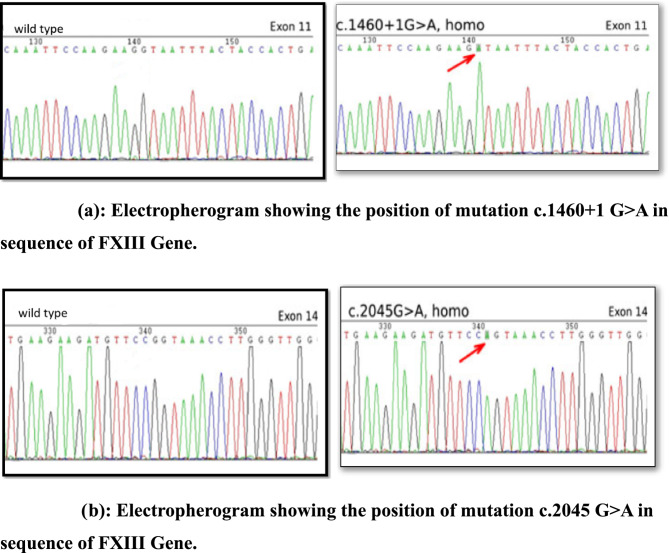
**a** Electropherogram showing the position of mutation c.1460+1 G>A in sequence of FXIII Gene. **b **Electropherogram showing the position of mutation c.2045 G>A in sequence of FXIII Gene

Gene Sequencing of the whole F13 gene was done by using Big dye terminator cycle sequencing kit (version 3.1) to identify mutations which are responsible for the deficiency of FXIII. In our study, we identified two mutations in FXIII gene which are IVS11 (+1) G>A in Exon 11 and c.2045G>A in Exon 14. (Figure 10a, 10b)

## Discussion

FXIII activity is emphasized in the study as a reliable biomarker for distinguishing carriers from non-carriers. These groups’ unique biochemical profiles can help with genetic counselling by allowing for the accurate identification of those who are at risk.

Finding carriers in Factor XIII (FXIII) deficient families is crucial for enhancing clinical care, enabling genetic counselling, and guaranteeing prompt action. The purpose of this study was to assess the role of clinical and demographic characteristics, namely FXIII activity and bleeding scores, in differentiating carriers from non-carriers. The results lay the groundwork for enhancing clinical results and diagnostic precision by delivering vital insights into carrier identification techniques [8].

This study’s intergenerational design included 187 participants (aged 1–85 years), enabling novel examination of FXIII activity and bleeding tendencies across generations. The mean age of 27.31 years reflects the target demographic for carrier identification and reproductive decision-making [7]. A useful viewpoint on inheritance patterns and the possibility of early diagnosis is offered by the inclusion of several generations. Early genetic counselling and therapeutic intervention may help younger members of impacted families and lessen the long-term effects of FXIII deficiency. Elderly family members, especially those who are asymptomatic carriers with borderline FXIII activity levels, offer vital information on the disease’s natural course and the function of compensatory mechanisms [10].

With a range of 1 to 19, the bleeding score analysis shows a mean score of 4.092, demonstrating substantial variation in bleeding tendencies across family members. These results highlight the fact that whereas most people only have little bleeding, others suffer from serious problems. This heterogeneity most likely reflects variations in FXIII activity levels as well as other moderating variables, such as lifestyle impacts or underlying clotting disorders [11].

There appears to be some stability in bleeding tendencies, as indicated by the moderate standard deviation of 2.6554, which might be related to hereditary patterns within families. Nonetheless, the necessity of customized evaluations is highlighted by the existence of outliers with high bleeding scores. These outliers may be people whose symptoms are being exacerbated by environmental variables, comorbidities, or compound deficits [12, 13].

It is impossible to overestimate the clinical significance of bleeding ratings as they provide a useful method for identifying possible carriers and ranking those who should have further testing. To determine carrier status and direct therapeutic options, a high bleeding score may lead to additional research, such as genetic testing and FXIII activity assays [14].

With mean activity levels of 49.96% and 100.95%, respectively, the examination of FXIII activity levels clearly distinguishes carriers from non-carriers. This noteworthy distinction demonstrates the value of FXIII activity as a trustworthy biomarker for carrier identification. The creation of diagnostic thresholds for carrier status may be aided by the more consistent deficit pattern shown by the decreased variability in FXIII activity among carriers (standard deviation of 26.27%) as opposed to non-carriers (79.22%) [15].

The results are consistent with the established pathophysiology of FXIII deficiency, which is characterized by heterozygous carriers exhibiting intermediate activity levels as a result of decreased FXIII-A or FXIII-B subunit production or function. In contrast, non-carriers exhibit normal or increased levels of activity, indicating that they are not genetically impaired [16].

Targeted carrier screening is made possible by this biochemical difference, especially in environments with limited resources where genetic testing might not be easily accessible [17].

The statistical significance of the differences in FXIII activity between carriers and non-carriers is confirmed by the one-way ANOVA findings (*F* = 19.44, *p* =.000). This supports the ability of FXIII activity tests to differentiate between these groups. The significant variation across groups (92,736.42) as opposed to within groups (849,043.64) highlights how reliable FXIII activity is as a marker for carrier identification [18].

The predictive usefulness of the FXIII activity and bleeding scores is further confirmed by logistic regression analysis. With a Nagelkerke R Square of 1.000 and a perfect match (−2 Log Likelihood = 0.000), the final model classified carriers and non-carriers with 100% accuracy. These results suggest that FXIII activity and bleeding scores together offer a thorough method of carrier identification that has both sensitivity and specificity [17].

Clinical practice and genetic counselling will be significantly impacted by the capacity to accurately identify carriers. Proactive steps including reproductive planning, prenatal diagnosis, and cascade testing of at-risk family members are made possible by early carrier discovery. Carrier identification can help guide decisions about anticoagulant usage, surgical planning, and other procedures that may increase the risk of bleeding in families with a documented history of FXIII deficiency [6]. Particularly in environments with limited resources, the incorporation of FXIII activity tests into standard clinical practice offers a financially viable substitute for genetic testing. The results of this study indicate that FXIII activity tests can be a useful first screening method, even if genetic testing is still the gold standard for carrier identification. Integrating clinical and biochemical data improves diagnosis precision and lessens the need for invasive or costly procedure [19]. Notwithstanding the encouraging results, several issues need to be resolved to maximize carrier identification tactics. The significant standard deviation (79.22%) of FXIII activity among non-carriers emphasizes the impact of confounding variables as age, sex, and comorbidities. To clarify these variables and create standardized reference ranges for FXIII activity based on clinical and demographic traits, more investigation is required.

Furthermore, because bleeding ratings are frequently derived from professional observations or self-reported symptoms, their use raises the possibility of subjectivity. Future research may benefit from increased consistency and reliability thanks to standardized bleeding assessment instruments like the International Society on Thrombosis and Haemostasis (ISTH) Bleeding Assessment Tool [9]

The study’s conclusions have wider public health ramifications, especially in areas where FXIII deficiency is very prevalent. Programs to identify carriers can greatly lessen the burden of undetected or improperly treated bleeding diseases, enhancing the lives of those who are impacted and their family. Campaigns to raise awareness, provider training, and the construction of diagnostic centres to aid in carrier identification should be the top priorities of public health activities [6, 11].

## Conclusion

The study’s findings highlight the critical role that FXIII activity and bleeding scores play in identifying carriers within FXIII-deficient families. A dependable, affordable, and easily accessible method of enhancing patient care and genetic counselling is provided by the incorporation of these diagnostic characteristics into clinical practice.

This study advances the diagnostic and treatment approaches for FXIII deficiency by tackling the difficulties and utilizing the knowledge acquired. In order to lessen the burden of bleeding disorders and enhance the quality of life for those who are impacted and their families, the results highlight the significance of early diagnosis, tailored treatment, and public health campaigns.

The ultimate objective is to guarantee that every person at risk for FXIII insufficiency has access to prompt and precise diagnosis, all-encompassing care, and the assistance required to live a healthy, satisfying life. The medical community can accomplish this aim and change the face of FXIII deficiency management globally with sustained research, cooperation, and innovation.

Despite the promising findings, several challenges remain that must be addressed to enhance carrier identification strategies. The variability in bleeding scores often influenced by subjective interpretation and environmental factors underscores the need for standardized assessment methods. Additionally, further investigation is required to minimize diagnostic inaccuracies related to the influence of coexisting medical conditions on FXIII activity levels.

The generalizability of the study’s outcomes is limited by its focus on a single community. Broader insights into FXIII deficiency and its clinical manifestations could be achieved by extending the research to include individuals from more diverse genetic backgrounds.

### Limitations of the study

This study provides important insights into identifying carriers of FXIII deficiency but has several limitations. FXIII levels may be affected by environmental factors, comorbidities, and lab variability, and limited access to genetic testing hindered definitive diagnosis in some cases. The restricted geographic population and cross-sectional design limit generalizability and the ability to track changes over time. Conventional statistical methods may have missed complex relationships, which could be addressed using advanced models in future research. Additionally, the study did not explore the psychosocial impact of carrier status. Addressing these gaps can improve diagnostic accuracy, broaden applicability, and support more holistic care.

## Data Availability

No datasets were generated or analysed during the current study.

## Abbreviations

FXIII: Factor XIII
EDTA: Ethylenediaminetetraacetic acid
G: Guanine
A: Adenine
DNA: Deoxyribonucleic Acid
CBC: Complete Blood Count
PT: Prothrombin Time
aPTT: Activated Partial Thromboplastin time
PPP: Platelet Poor Plasma
ELISA: Enzyme Linked Immunosorbent Assay
